# Neuronal Cholesterol Accumulation Induced by Cyp46a1 Down-Regulation in Mouse Hippocampus Disrupts Brain Lipid Homeostasis

**DOI:** 10.3389/fnmol.2017.00211

**Published:** 2017-07-11

**Authors:** Sophie Ayciriex, Fathia Djelti, Sandro Alves, Anne Regazzetti, Mathieu Gaudin, Jennifer Varin, Dominique Langui, Ivan Bièche, Eloise Hudry, Delphine Dargère, Patrick Aubourg, Nicolas Auzeil, Olivier Laprévote, Nathalie Cartier

**Affiliations:** ^1^UMR Centre National de la Recherche Scientifique 8638 COMETE, Sorbonne Paris Cité, Faculté des Sciences Pharmaceutiques et Biologiques, Université Paris Descartes Paris, France; ^2^Institut National de la Santé et de la Recherche Médicale U1169, CHU Bicêtre Paris Sud Le Kremlin-Bicêtre, France; ^3^CEA Fontenay aux Roses Fontenay aux Roses, France; ^4^Division Métabolisme, Technologie Servier Orléans, France; ^5^Génétique, Physiopathologie et Approches Thérapeutiques des Maladies Héréditaires du Système Nerveux, EA7331, Faculté des Sciences Pharmaceutiques et Biologiques, Université Paris Descartes Sorbonne Paris Cité, Paris, France; ^6^Plate-forme d'Imagerie Cellulaire Pitié Salpêtrière, Hôpital Pitié-Salpêtrière Paris, France; ^7^Alzheimer's Disease Research Laboratory, Department of Neurology, Massachusetts General Hospital Charlestown, MA, United States; ^8^Service de Toxicologie Biologique, Hôpital Lariboisière Paris, France

**Keywords:** neurodegeneration, gene silencing, Cyp46a1, cholesterol, lipidomics, lipid dysregulation, ER stress

## Abstract

Impairment in cholesterol metabolism is associated with many neurodegenerative disorders including Alzheimer's disease (AD). However, the lipid alterations underlying neurodegeneration and the connection between altered cholesterol levels and AD remains not fully understood. We recently showed that cholesterol accumulation in hippocampal neurons, induced by silencing *Cyp46a1* gene expression, leads to neurodegeneration with a progressive neuronal loss associated with AD-like phenotype in wild-type mice. We used a targeted and non-targeted lipidomics approach by liquid chromatography coupled to high-resolution mass spectrometry to further characterize lipid modifications associated to neurodegeneration and cholesterol accumulation induced by CYP46A1 inhibition. Hippocampus lipidome of normal mice was profiled 4 weeks after cholesterol accumulation due to *Cyp46a1* gene expression down-regulation at the onset of neurodegeneration. We showed that major membrane lipids, sphingolipids and specific enzymes involved in phosphatidylcholine and sphingolipid metabolism, were rapidly increased in the hippocampus of AAV-shCYP46A1 injected mice. This lipid accumulation was associated with alterations in the lysosomal cargoe, accumulation of phagolysosomes and impairment of endosome-lysosome trafficking. Altogether, we demonstrated that inhibition of cholesterol 24-hydroxylase, key enzyme of cholesterol metabolism leads to a complex dysregulation of lipid homeostasis. Our results contribute to dissect the potential role of lipids in severe neurodegenerative diseases like AD.

## Introduction

Mammalian brain, most cholesterol-rich organ contains 10-fold more cholesterol than any other organ (Dietschy and Turley, 2004; Bjorkhem, 2006; Bjorkhem et al., 2010). The capacity of the brain to store such a large amount of cholesterol indicates that this sterol plays a central role in the development and function of the brain. About 70% of cholesterol is found in myelin. The remaining 30% of brain cholesterol is distributed between glial cells (20%) and neurons (10%) (Pfrieger, 2003; Bjorkhem and Meaney, 2004; Dietschy and Turley, 2004; Vance et al., 2005; Bjorkhem et al., 2010). Cholesterol is involved in the formation of axons and dendrites during development, neuronal repair and remodeling, formation of new synapses, learning, and memory (de Chaves et al., 1997; Xu et al., 1998; Mahley and Rall, 2000; Mauch et al., 2001). Brain cholesterol metabolism is tightly regulated. The cholesterol abundance in the central nervous system depends primarily on local synthesis and efflux. Cholesterol does not freely cross the intact blood-brain barrier (BBB) and nearly all cholesterol in the adult brain is formed by *in situ* synthesis (Turley et al., 1996; Vance et al., 2005; Bjorkhem et al., 2010). One of the mechanisms for cholesterol clearance from the brain is its conversion into an hydroxylated metabolite, the 24(*S*)-hydroxycholesterol (24-OHC), which is able to cross the BBB and enter the circulation to the liver to be further metabolized to bile acids (Lund et al., 2003; Bjorkhem, 2006; Russell et al., 2009). This conversion is catalyzed by an enzyme cytochrome P450, the cholesterol 24-hydroxylase, also called CYP46A1, mainly expressed by neuronal cells (Lutjohann et al., 1996; Lund et al., 1999). This conversion represents one of the most important mechanisms for cholesterol clearance from the brain (Bjorkhem, 2006; Russell et al., 2009).

Several clinical and biochemical studies have suggested that cholesterol imbalance in the brain may be associated to neurodegenerative disorders, such as Alzheimer's disease (AD), Niemann–Pick disease type C, Huntington's disease and Parkinson's disease (Puglielli et al., 2003; Wolozin, 2004; Valenza et al., 2005, 2010; Liu et al., 2010; Cheng et al., 2011; Meljon et al., 2013). In AD, cholesterol affects amyloid processing pathway (Aβ generation and aggregation). Aβ is produced by sequential cleavage of the amyloid precursor protein (APP) by β-secretase (BACE1) and γ-secretase. Cholesterol promotes the co-clustering of APP and BACE1 in lipid raft domains leading to Aβ production and accumulation (Marquer et al., 2011; Ayciriex et al., 2016). Cholesterol seems to play a pivotal role in the neurodegenerative process of AD.

We recently developed an *in vivo* model to study the consequences of neuronal cholesterol accumulation in normal mice (Djelti et al., 2015). We delivered a *Cyp46a1* specific short-hairpin RNA (shCYP46A1) in C57Bl/6 mice hippocampus using stereotactic injection of an adeno-associated viral vector serotype 5 (AAV5). Efficient inhibition of *Cyp46a1* gene expression in hippocampus of injected mice led to an accumulation of cholesterol in neurons 3 and 4 weeks after injection (Chali et al., 2015; Djelti et al., 2015). This cholesterol accumulation was accompanied by marked changes in endosomes, Aβ peptides production, tau phosphorylation and endoplasmic reticulum (ER) stress. This cascade of events finally triggered apoptotic cell death, 4 weeks after injection. These processes induced cognitive impairment and hippocampal atrophy, 12 weeks after injection. All these observations closely mimic the pathological hallmarks of AD. Down-regulation of *Cyp46a1* gene expression in APP23 mice strongly aggravated the increase of Aβ peptides production, and induced phosphorylation of tau protein and neuronal death (Djelti et al., 2015). These results strongly suggest that accumulation of cholesterol plays a pivotal role in neurodegenerative process and AD.

Altered lipid homeostasis alteration in the brain could be a risk factor for the many types of neurodegenerative disorders, including Alzheimer's disease, Huntington's disease, and Parkinson's disease. These neurodegenerative disorders are marked by extensive neuronal apoptosis, gliosis (Han, 2005; Fan et al., 2013; Yadav and Tiwari, 2014). Several lipidomics studies have shown lipid homeostasis modifications in AD brain tissue (Han, 2005; Di Paolo and Kim, 2011; Chan et al., 2012; Panchal et al., 2014). Changes in cholesterol, sulfatide, ceramide, galactosylceramide, and plasmalogens are highlighted in brain of AD patients (Han et al., 2001, 2002; Cutler et al., 2004; Han, 2005). However, the connection between lipids alterations, cholesterol accumulation and neurodegenerative process in AD remains not fully understood.

In the present work, we investigated the consequences of *Cyp46a1* gene expression inhibition and neuronal cholesterol accumulation on hippocampal lipidome, 4 weeks after AAV-shCYP46A1 injection a time corresponding to the onset of neuronal loss. First, the sterol and oxysterol contents were analyzed by a targeted approach combining ultra-performance liquid chromatography (UPLC) and high-resolution mass spectrometry (Ayciriex et al., 2012). Second, an untargeted lipidomics approach without *a priori* knowledge was performed using the same analytical platform to monitor lipid perturbations. Our data showed that *Cyp46a1* gene silencing lead to a decrease of 24(*S*)-hydroxycholesterol, 25-hydroxycholesterol and to an increase of cholesterol content. The major membrane lipids, such as phosphatidylethanolamine (PE) and phosphatidylcholine (PC) were increased together with sphingolipids including sulfatides, ceramides (Cer), and glucosylceramides (GlcCer), and gangliosides GM1. Diacylglycerols (DAG) with long and unsaturated fatty acid moieties were also increased. Expression of genes encoding specific enzymes involved in phosphatidylcholine and sphingolipid metabolism was also increased in response to cholesterol overload induced by *Cyp46a1* gene silencing. In parallel, we highlighted lysosomal dysfunction characterized by an increase of lysosomes number and the presence of phagolysosomes in the hippocampus of AAV-shCYP46A1 injected mice. Our results further contribute to understand the mechanisms of neurodegeneration mediated by cholesterol accumulation in neurons following impairment of *Cyp46a1* gene expression.

## Materials and methods

### Chemicals and reagents

Triethylamine, 4-(dimethylamino)phenyl isocyanate, formic acid and butylated hydroxytoluene (BHT) were obtained from Sigma-Aldrich (Saint-Quentin Fallavier, France). Hexane and dichloromethane were obtained from CarloErbaReactifs SDS (Val-de-Reuil, France). Acetonitrile, methanol, isopropanol were of LC-MS grade (J.T. Baker, Phillipsburg, NJ, USA). 24(*R/S*)-hydroxycholesterol d6 [26,26,26,27,27,27-hexadeuterocholest-5-ene-3ß,24-diol], cholesterol d7 [cholest-5-en-3ß-ol(d7)], 1,2-diheptadecanoyl-*sn*-glycero-3-phosphate, PA(17:0/17:0), 1,2-diheptadecanoyl-*sn*-glycero-3-phospho-L-serine, PS (17:0/17:0), 1,2-diheptadecanoyl-*sn*-glycero-3-phosphoethanolamine, PE(17:0/17:0), 1,2-diheptadecanoyl-*sn*-glycero-3-phosphocholine, PC(17:0/17:0), 1,2-diheptadecanoyl-*sn*-glycero-3-phospho-(1′-rac-glycerol), PG(17:0/17:0), 1,2-diheptadecanoyl-*sn*-glycerol, DG (17:0/17:0/0:0) and *N*-heptadecanoyl-D-*erythro*-sphingosine, Cer(d18:1(4E)/17:0) were obtained from Avanti Polar Lipids, Inc. (Alabaster, AL, USA).

### AAV plasmid design and vectors production

The short-hairpin (sh) RNA, against the sequence specific of *Cyp46a1* gene with the promoter U6, was amplified and cloned into AAV5 vector (Djelti et al., 2015). In parallel a scramble sequence that contained no similarity to the endogenous mRNA, was used as a negative control. AAV5 vector also contained eGFP reporter sequence under the control of the PGK promoter. AAV5 viral stocks were produced by transient transfection of 293T cells with the respective viral vector and the subsequent purification of the cell culture supernatant by a caesium-chloride ultracentrifugation gradient to yield titers of 4 to 9 × 101^2^ Vg/mL (Sevin et al., 2006). The vectors, used in this study, were named AAV-scramble (control) or AAV-shCYP46A1 (Supplementary Table S1).

### Animals and intracerebral injections of AAVs

Twelve weeks-old female wild-type C57Bl/6 mice (Janvier, France) were housed in pathogen-free conditions with a 12 h light/dark cycle (average weight 20–25 g). These experiments were carried out in strict accordance with the recommendations in the Guide for the Care and Use of Laboratory Animals of the National Institutes of Health (NIH publication no.85-24) and the European Committee Council Directive (86/89/EEC). The protocol for animal experiments was approved by the Committee on the Ethics of Animal Experiments of the Ministere de l'Enseignement Superieur et de la Recherche (Permit Number: B92-032-02).

Mice were anesthetized by intraperitoneal injection of ketamine (80 mg/kg) and xylazine (50 mg/kg) and placed on a stereotaxic frame (David Kopf Instruments, Tujunga, CA). All efforts were made to minimize suffering. The AAV5-vector was injected in one hippocampus (*stratum lacunosum moleculare*) with 2 μl of viral preparation (2 × 10^9^ vg) using a 30-gauge needle attached to a 10-μL Hamilton syringe (Hamilton Medical, Reno, NV) at a rate of 0.2 μL/min. Stereotactic coordinates of injection sites from bregma were anterior-posterior: -2 mm; medial-lateral: −1.2 mm; dorsal-ventral: −2 mm. C57Bl/6 mice were sacrificed at 3 and 4 weeks after injection for gene expression, immunofluorescence, electron microscopy studies (*n* = 5 mice per vector, per time, per analysis). The lipid analyses were undertaken only at 4 weeks after injection, at the onset of neuronal loss. Anesthetized animals were transcardially perfused with PBS1X (Life technologies). Dissected hippocampi were frozen in liquid nitrogen and conserved at −80°C.

### Gene expression analysis by qPCR

Dissected hippocampi were snap frozen in liquid nitrogen and ground into powder for total mRNA extration using the RNAble kit (Eurobio laboratories, Les Ulis France). Quantitative real-time PCR (qPCR) analysis was carried out using the ABI Prism® 7900HT sequence detection system (Applied Biosystems, Foster City, CA, USA) as described previously (Bieche et al., 2004). As an endogenous RNA control, transcripts of the TATA-box binding protein gene (TBP) were measured. Primers for TBP and the other gene were chosen with the assistance of the Oligo 5.0 computer program (National Biosciences, Plymouth, MN) (Supplementary Table S2). Target transcript levels (N_target_) were normalized according to TBP content and then to a basal mRNA level following the equation N_target_ = 2.δ^Ct^, where δ^Ct^ is the Ct value of the target gene after subtraction of Ct for the TBP gene.

### Lipid sample preparation

Hippocampus tissues were homogenized in cold water containing 0.1% BHT with a Precellys®24-Dual (Precellys) during 15 s at 5,000 rpm. The brain tissue homogenization was performed in 2 mL tube prefilled with 1.4 mm ceramic beads. 5 μL of homogenate were used for protein estimation using the BCA Protein Assay Kit-Reducing Agent Compatible (Pierce). The rest of the homogenate were spiked with an internal standard mix composed of PA(17:0/17:0), PS(17:0/17:0), PE(17:0/17:0), PC(17:0/17:0), PG(17:0/17:0), DG(17:0/17:0/0:0), and Cer(d18:1(4E)/17:0)] at a concentration of 6.25 μM prior to lipid extraction. Lipids were extracted with hexane/methanol mixture (3:1, *v/v*) by mechanical shaking for 1 h, at room temperature in 13 × 100 mm glass tubes with PTFE-lined caps. The extracts were centrifuged at 2,500 rpm for 10 min to achieve phase separation. The organic phase (upper layer) was collected and washed with 600 μL water for 10 min on a mechanical shaker. The upper phases were pooled and divided into two fractions: one fraction for cholesterol and oxysterol analysis (fraction F1) and the other for global lipidomics analysis (fraction F2). All fractions were dried under nitrogen gas.

#### Cholesterol and oxysterols analysis

F1 was resuspended in 200 μL of methanol and spiked with 24(*R/S*)-hydroxycholesterol (*d6*) (20 ng) and cholesterol (*d7*) (20 ng) used as an internal standard for oxysterols and cholesterol quantification, respectively. Oxysterols and cholesterol were derivatized with 4-(dimethylamino)phenyl isocyanate and analyzed by UPLC-ESI-HRMS according to the procedure previously described in Ayciriex et al. (2012). Oxysterols and cholesterol contained in hippocampi was in the range of ng/mg and μg/mg of proteins, respectively.

#### Lipidomics analysis of mice hippocampus by UPLC-ESI-QTOF-MS^E^

F2 was resuspended in 200 μL of acetonitrile/isopropanol mixture (1:1, *v/v*). Quality control (QC) samples were prepared by combining 20 μL of each lipid extracts. After evaporation, QC samples were reconstituted in a small volume of acetonitrile/isopropanol mixture and further diluted to one-third and one-sixth diluted QC samples. A 3 μL aliquot of QC and samples were injected into ACQUITY UPLC® system coupled to an ESI-QTOF-MS (SYNAPT® G2 High Definition MS™ mass spectrometer, Waters, UK). Lipids were separated on an ACQUITY UPLC® HSS T3 1.8 μm column (2.1 × 100 mm) thermostated at 50°C at a flow rate of 0.40 mL.min^−1^ with acetonitrile and water with 10 mM ammonium acetate (40:60, *v/v*) as eluent A and acetonitrile/isopropanol (10:90, *v/v* containing 10 mM ammonium acetate as eluent B (Castro-Perez et al., 2010). Data were collected separately both in positive (ESI^+^) and negative (ESI^−^) ion mode. ESI source parameters were as follows: source temperature 120°C, desolvation temperature 450°C, cone gas flow 20 L/h, desolvation gas flow 800 L/h, capillary voltage 2,400 V (ESI^−^), 3,000 V (ESI^+^), cone voltage 45 V (ESI^−^), and 30 V for (ESI^+^). The mass spectrometer was operated in the MS^E^ mode of acquisition for both polarities. Two independent acquisitions functions are automatically created in MS^E^ mode. The first function set at 5 eV collects data on unfragmented ions while the second function collects fragmentation data by using a collision energy ramp from 20 to 50 eV. In addition MS^2^ experiments were performed to confirm structural identification of ions of interest.

Centroided accurate mass spectra were acquired over the *m/z* range 50–1,000 with a scan time of 0.1 s and an interscan delay of 0.01 s using a target mass resolution of 21,500 (Full width at half maximum, FWHM as defined at *m/z* 500). Mass was corrected during acquisition using a 2 ng/μL solution of leucine enkephalin in acetonitrile/water (1:1, *v/v*) as an external reference (Lock-SprayTM), with an analyte-to-reference scan ratio of 20:1. Ten QC samples were injected at the beginning of the run in order to condition the column and also injected regularly throughout the analytical batch to ensure system stability and robustness of the method (Want et al., 2013). No major drift in signal response during the analytical run was observed. Each sample was injected in triplicate. In addition, to avoid any bias related to the order of injection, sample run order was set orthogonal to experimental design (Want et al., 2010).

#### Data processing

Raw data files (.raw format) acquired on the UPLC-MS^E^ platform were converted to NetCDF format (Waters Databridge software). Deconvolution of data was performed on XCMS online software with parameters settings suitable for high resolution UPLC-MS data acquired in centroid mode (Smith et al., 2006; Tautenhahn et al., 2012b). Subsequent to data pre-processing, a table listing peak intensity associated to a unique retention time and *m/z* as identifiers (tR_*m/z* data pairs) vs. samples, blank and QC, was generated in both ionization modes. After normalization of the variables to the total intensity and protein concentration, the final dataset was analyzed by multivariate data analysis. The discriminating lipid species identified after supervised analyses were quantified by normalizing the intensities of their peaks to the intensity of the peaks of the corresponding internal standards spiked into the sample prior to lipid extraction and expressed as pmol/mg proteins.

#### Multivariate statistical analysis

Data was pareto-scaled and subjected to principal component analysis (PCA) and orthogonal partial least square discriminant analysis (OPLS-DA) using SIMCA software (v. 13, MKS Umetrics AB, Sweden) (Wiklund et al., 2008).

#### Lipid nomenclature

The abbreviations used were according to LipidMaps recommendations (Fahy et al., 2005, 2009). Glycerophospholipids and diacylglycerol were annotated as <lipid subclass>(<total fatty acyl chain length>: <total number of unsaturated bonds>). Sphingolipids were annotated as <lipid subclass>(<sphingoid base residue>/< fatty acyl residue>). Sulfoglycosphingolipids or sulfatides were annotated according to their common name described in Lipid Maps structure database. Lipid identification Lipid species annotation was performed through the online database LIPID MAPS (http://www.lipidmaps.org/) and METLIN Metabolite Database (metlin.scripps.edu) using 5 ppm of mass accuracy as a tolerance window (Sud et al., 2007; Tautenhahn et al., 2012a). Lipid species structure was confirmed by MS^E^ fragmentation spectra analysis and selected MS^2^ experiments. MS^2^ data analysis highlights product ions, which are characteristic of lipid class and can serve to discriminate between database hits. The analytical reliability of each ion signal of interest was checked by calculating its relative standard deviation (% r.s.d.) in QC samples (Chan et al., 2011). Electron Microscopy 4 weeks after injection, C57Bl/6 mice were lethally anesthetized and perfused with 4% paraformaldehyde and 2.5% glutaraldehyde in phosphate buffer (PB) 0.12 M pH 7.4. Brains were post-fixed in 2.5% glutaraldehyde in PB. The eGFP fluorescent region from the hippocampus of C57Bl/6 mice injected with AAV−scramble or AAV-shCYP46A1 was excised under a fluorescence dissecting binocular microscope (Leica Z16 APO). Sections of 50 μm were cut with a vibratome and post-fixed in 1% osmium tetroxide for 30 min, rinsed in PB, dehydrated in a graded series of ethanol solutions (75, 80, 90, and 100%), infiltrated with EponTM812, placed in molds and the resin was cured at 60°C in a dry oven during 48 h. Hippocampal semi-thin sections, 0.5 μm thick, obtained with a Leica UC7 ultramicrotome, were stained with a 1% toluidine solution. Ultra-thin sections (90 nm) were cut, counterstained with uranyl acetate (2%) and Reynolds lead citrate (Reynolds, 1963) and observed with a Hitachi HT7700 electron microscope, operating at 80 kV. Pictures (2,048 × 2,048 pixels) were taken with an AMT41B (size of the pixel 7.4 × 7.4 μm).

### Immunohistochemical analysis

Brain tissue obtained at 4 weeks after AAV5-vector injection (scramble or shCYP46A1) was used for anatomical experiments (*n* = 5 mice from by vector). Animals were lethally anesthetized and perfused transcardially with PBS1X (Life technologies™, France). After a post-fixation in PBS1X, 4% paraformaldehyde in for 24 h, the brain was embedded in paraffin. Sections of 6 μm thickness were sequentially (i) deparaffined in xylene, (ii) rehydrated in ethanol, (iii) permeabilized in PBS1X, 0.1% triton X100, (iv) blocked in PBS1X, 0.1% triton X100, 5% normal goat serum and incubated at 4°C for 30 min, in PBS1X, 0.1% triton X100, 5% normal goat serum with an antibody against anti-LAMP1 coupled to biotin for 1 h (1/200, Abcys). Appropriate secondary antibodies were applied 1 h at room temperature. The slides were then mounted in Vectashield® mounting medium containing DAPI (Vectorlabs) and examined by fluorescence or confocal microscopy.

For fluorescence microscopy, images were taken with a Nikon microscope (Eclipse 800) and a digital QIMAGING camera (CCD QICAM cooled plus RGB filter pixel 4.65 × 4.65 μm). Control and test slices were processed the same day and under the same condition. Images were acquired by confocal microscopy using Zeiss LSM 510 Meta confocal laser microscope with a Plan apochromat 63x/1.4 numeric aperture oil immersion objective using the LSM 10v4.osp2. The number of LAMP-1 positive lysosomes in pyramidal neurons of CA3a region was measured in 60 cells per mouse (*n* = 5 mice per vector, six sections) using Image J software.

### Electron microscopy

Four weeks after injection, C57Bl/6 mice were lethally anesthetized and perfused with 4% paraformaldehyde and 2.5% glutaraldehyde in phosphate buffer (PB) 0.12 M pH 7.4. Brains were post-fixed in 2.5% glutaraldehyde in PB. The eGFP fluorescent region from the hippocampus of C57Bl/6 mice injected with AAV5-scramble or AAV5-shCYP46A1 was excised under a fluorescence dissecting binocular microscope (Leica Z16 APO). Sections of 50 μm were cut with a vibratome and post-fixed in 1% osmium tetroxide for 30 min, rinsed in PB, dehydrated in a graded series of ethanol solutions (75, 80, 90, and 100%), infiltrated with EponTM812, placed in molds and the resin was cured at 60°C in a dry oven during 48 h. Hippocampal semi-thin sections, 0.5 μm thick, obtained with a Leica UC7 ultramicrotome, were stained with a 1% toluidine solution. Ultra-thin sections (90 nm) were cut, counterstained with uranyl acetate (2%) and Reynolds lead citrate (Reynolds, 1963), and observed with a Hitachi HT7700 electron microscope, operating at 80 kV. Pictures (2,048 × 2,048 pixels) were taken with an AMT41B (size of the pixel 7.4 × 7.4 μm).

### Statistical analysis

Experimental values are presented as mean ± SEM. Statistical analysis was performed using GraphPad Prism software version 5.0 (San Diego, CA). The significance of cholesterol and oxysterols concentration was determined using Mann-Whitney test with Bonferroni correction. Unpaired *t*-test was performed for gene expression analysis. In all cases, ^*^p < 0.05, ^**^p < 0.01, and ^***^p < 0.001.

## Results

### Effects of *Cyp46a1* silencing on the hippocampus steroidome

**Figure 1:**
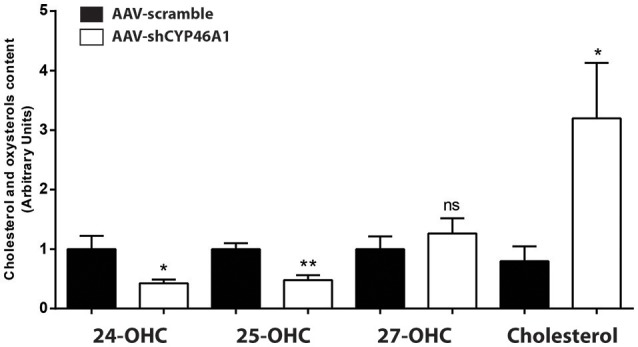
Measurement of cholesterol and oxysterols content 4 weeks after hippocampal injection of AAV-shCYP46A1 vector. AAV-scramble (control) or AAV-shCYP46A1 vector was injected in the *stratum lacunosum moleculare* of hippocampus in C57BL/6 mice. Sterols were extracted, derivatized and analyzed by UPLC-ESI-Q-TOF in MS scan mode. Cholesterol, 24-, 25-, and 27-hydroxycholesterol contents were quantified and normalized to AAV-scramble content (*n* = 5 mice). Unpaired *t*-test was performed. ^*^ *P* < 0.05, ^**^P < 0.01; ns, non-significant.

AAV-shCYP46A1 and AAV-scramble (control) vectors were injected in the *stratum lacunosum moleculare* region of the hippocampus of 12 weeks old C57Bl/6 mice (*n* = 5 mice per time per vector). The viral vectors were engineered to express the green fluorescent reporter protein (eGFP) to allow the detection of transduced cells. Three weeks after vector injection, eGFP expression was mainly detected in the neurons of CA3a region of the injected hippocampus (Djelti et al., 2015). Real-time qPCR experiments confirmed that the injection of AAV-shCYP46A1 induced 70% inhibition of *Cyp46a1* gene expression, 3 and 4 weeks after injection (Djelti et al., 2015). Cholesterol-24(*S*)-hydroxylase encoded by *Cyp46a1* gene converts the cholesterol into 24(*S*)-hydroxycholesterol and to a lesser extend into 25-hydroxycholesterol (Lund et al., 1999). The inhibition of *Cyp46a1* gene expression leads to a decrease of 24(*S*)-hydroxycholesterol content by 57% and of 25-hydroxycholesterol content by 52%, 4 weeks after injection (Figure 1). As expected, 27-hydroxycholesterol content, which is not produced by cholesterol-24(*S*)-hydroxylase, was not modified. A 3-fold increase of cholesterol content was observed in the hippocampus of mice injected with AAV-shCYP46A1 after 4 weeks. No change in other sterols or oxidative product of cholesterol, such as desmosterol or 5α,6α-epoxycholesterol was observed (Supplementary Figure 1). These results indicate that the injection of AAV-shCYP46A1 induced a strong reduction of cholesterol-24(*S*)-hydroxylase activity leading to a decreased production of 24-(*S*) hydroxycholesterol and to an accumulation of cholesterol.

### Cholesterol accumulation was associated with an increase in phospholipids and sphingolipid species

#### Multivariate data analyses

**Figure 2:**
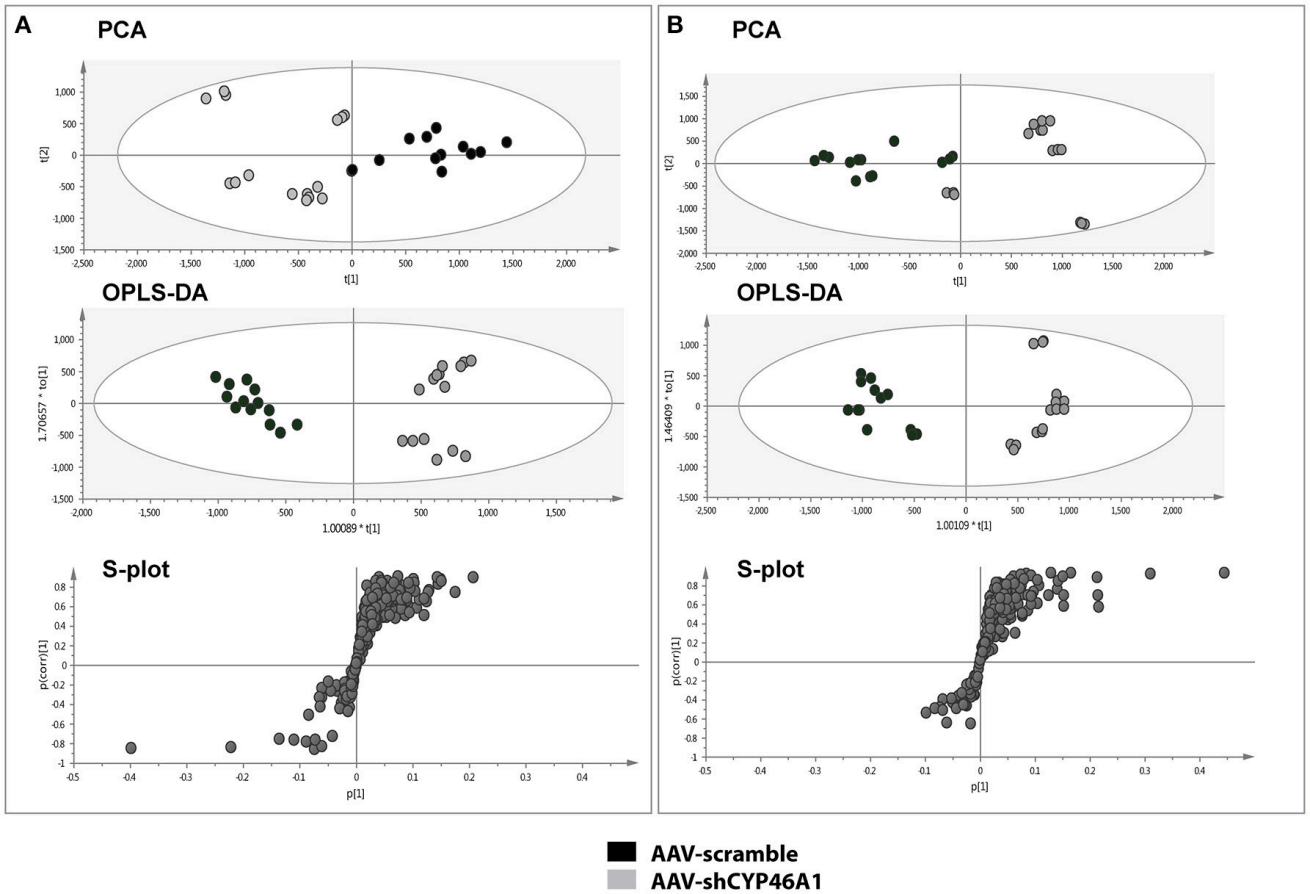
Multivariate data analysis of AAV-scramble and AAV-shCYP46A1 hippocampus lipid data. After extraction, the lipid content of AAV-scramble (control) and AAV-shCYP46A1 hippocampus 4 weeks after injection were analyzed by UPLC-ESI-MS^E^. **(A)** PCA, OPLS-DA scores plots and OPLS-DA loadings S-plot for ESI^+^ mode. For the PCA score plot, the principal component 1 (87%) and principal component 2 (5%) account for 92% of the variance. For the OPLS-DA score plot: one orthogonal and two predictive components, R2X(cum) = 0.837, R2Y(cum) = 0.947, Q2(cum) = 0.927 with a *p*-value, *p* = 7.27e-11. S-plot from OPLS-DA analysis emphasizes variables that strongly contributed to the class separation between the two groups. **(B)** PCA, OPLS-DA scores plots and OPLS-DA loadings S-plot for ESI- mode. For the PCA score plot, the principal component 1 (87%) and principal component 2 (4%) account for 91% of the variance. For the OPLS-DA score plot: one orthogonal and two predictive components, R2X(cum) = 0.757, R2Y(cum) = 0.953, Q2(cum) = 0.905 927 with a *p*-value, *p* = 1.14e-9. S-plot from OPLS-DA analysis emphasizes variables that strongly contributed to the class separation between the two groups. AAV-scramble and AAV-shCYP46A1 are represented by black and gray dots, respectively. Details of variables are shown in Tables 1, 2.

To investigate the consequences of neuronal cholesterol accumulation on hippocampus lipidome, we performed an untargeted lipidomic analysis 4 weeks after AAV5-vector injection. The lipid contents of hippocampus tissue from control or AAV-shCYP46A1 mice, 4 weeks after injection, were analyzed by UPLC-ESI-MS^E^ in negative and positive ion mode (Supplementary Figure 2). First, UPLC-MS data set obtained from ESI^+^ and ESI^−^ were processed by XCMS software. This processing step generates a list of variables annotated with *t*R_*m/z* ions pairs. After data set normalization, the data analysis is followed by multivariate analysis to identify the most discriminating lipids between the two groups. To easily visualize any clustering between the different sample groups between AAV-scramble (control) and AAV-shCYP46A1 samples, an unsupervised analysis was performed. Indeed, the dimensionality of the listed variables was reduced by unsupervised PCA and a pareto scaling was applied to the data-sets obtained in positive and negative ionization modes. The two sample groups, AAV-scramble and AAV-shCYP46A1 exhibited a separation suggesting that cholesterol accumulation induced by *Cyp46a1* gene silencing led to differences in hippocampus lipidome (Figures 2A,B). To identify the variables contributing to the separation between the two sample groups, a supervised OPLS-DA was performed. The score plot of OPLS method shows an excellent group separation between the two sample groups (Figures 2A,B). The corresponding S-plot allowed the selection of discriminating variables. Only variables that exhibit a correlation p (corr) higher than 0.7 or lower than −0.7, were explored.

#### Lipid annotation and identification

The variables selected, characterized by *t*R_*m/z* ions pairs, were annotated using LipidMaps and Metlin online databases, according to the exact mass. Among 709 and 234 variables detected, respectively in ESI^−^ and ESI^+^, 22 lipid species in total were annotated (Tables 1, 2). These 22 lipids species in AAV-shCYP46A1 hippocampus were increased compared to AAV-scramble. No decrease of any lipid species was observed. The untargeted analysis revealed an increase in glycerophospholipids (PE, PE-P, PC) and glycerolipids (DG) and sphingolipids (Cer, GlcCer, sulfatide) in AAV-shCYP46A1 injected hippocampus (Tables 1, 2). Confirmation of lipid identity was achieved by the analysis of the spectra acquired in MS^E^ and MS^2^ modes.

**Table 1:** Discriminative lipid species whose detected levels in AAV-shCYP46A1 4 weeks after injection are higher than in the control (AAV-scramble) predicted by OPLS-DA obtained from positive ion mode data set.

***m/z* [M+H]^+^**	***t*_R_ (min)**	**Annotation**	**Fold change in level content (sh/scramble)**	**p (corr)**	**p (1)**	**CV% in QC**	**MS/MS (*m/z*)^#^**
706.53	5.72	PC 14:0/16:0	1.56	0.82	0.05	6	184.07–450.29–468.30–478.32–496.33
732.55	5.82	PC 16:0/16:1	1.49	0.89	0.09	3	184.07–313.27–476.31–478.32–496.33
760.58	6.33	PC 16:0/18:1	1.35	0.90	0.44	1	184.07–478.32–496.33–504.34–577.52
762.60	6.75	PC 16:0/18:0	1.24	0.73	0.14	2	184.07–478.32–496.33–506.36–524.37
786.60	6.41	PC 18:1/18:1	1.41	0.92	0.13	4	184.07-478.32-504.34-522.35-603.53
788.61	6.83	PC 18:0/18:1	1.37	0.69	0.22	2	184.07–339.29–504.34-506.36-522.35
806.56	5.81	PC 16:0/22:6	1.48	0.83	0.14	3	184.07-385.27-478.33–496.33–623.50
832.58	5.88	PC 18:1/22:6	1.61	0.81	0.06	10	184.07–385.27–504.34–550.32–649.52
634.54	7.08	DAG 36:4	1.64	0.78	0.04	4	ND
660.55	7.13	DAG 38:5	1.75	0.72	0.03	15	ND
662.57	7.51	DAG 38:4	1.69	0.78	0.11	8	ND

# *Indicates characteristic fragment ions*.

**Table 2:** Discriminative lipid species whose detected levels in AAV-shCYP46A1 4 weeks after injection are higher than in the control (AAV-scramble) predicted by OPLS-DA obtained from negative ion mode data set.

***m/z***	***t*_R_ (min)**	**Annotation**	**Fold change in level content (sh/scramble)**	**p (corr)**	**p (1)**	**CV% in QC**	**MS/MS (*m/z*)^#^**
646.61^a^	7.76	Cer d18:1/24:1	1.65	−0.81	−0.06	6	237.22–263.24–390.37–406.37–598.60–616.60
810.68^a^	7.67	GlcCer d18:1/24:0	1.62	−0.68	−0.09	5	237.22–263.24–392.38–408.37–600.60–648.63
808.66^a^	7.28	GlcCer d18:1/24:1	1.64	−0.69	−0.16	5	237.22–263.24–390.37–406.37–598.60–646.61
764.52^a^	6.06	PE 18:1/20:4	1.37	−0.65	−0.07	6	196.04–281.24–303.23–478.29
788.52^a^	5.95	PE 18:1/22:6	1.32	−0.75	−0.05	5	196.04–281.24–327.23–478.29
744.55^a^	6.88	PE 18:0/18:1	1.53	−0.65	−0.09	4	196.03–281.24–283.26
716.52^a^	5.77	PE 16:0/18:1	1.53	−0.74	−0.03	14	196.03–253.21–255.23–281.24–283.26
728.55^a^	7.14	PE P-18:0/18:1	1.86	−0.66	−0.12	4	196.03–281.24–339.29–392.29
806.54^a^	5.33	sulfatide C18:0	2.03	−0.82	−0.07	6	96.95–241–308.29–522.27–564.53–566.30
822.54^a^	5.21	sulfatide C18:0(OH)	2.08	−0.71	−0.04	10	96.96–241–324.29–522.27–540.28
886.60^b^	5.93	sulfatide C24:1(OH)	2.12	−0.72	−0.04	11	96.96–241–327.23–581.30–599.32

# *Indicates characteristic fragment ions*.

The identities of the glycerophospholipids PC, PE, and PE-plasmalogens (PE-P) were confirmed by monitoring their respective characteristic polar head group ions: m/z 184.07 in ESI^+^ (phosphocholine), m/z 196.03 in ESI-(glycerol phosphoethanolamine with a water loss) (Tables 1, 2).

The fatty acyl chains detected in negative ion mode enable to determine the molecular species, e.g., m/z 253.2 (FA 16:1), 255.2 (FA 16:0), 281.2 (FA 18:1), 283.2 (FA 18:0), 303.23 (FA 20:4), 327.2 (FA 22:6) (Table 2). PE(P-18:0/18:1) upon collision-induced dissociation (CID) exhibits fragment-ions at m/z 392.2 and 339.2, corresponding to the fatty alcohol species C18:0 at sn-1 position and the fatty acyl moiety (FA 18:1) at sn-2 position of the glycerol backbone (Zemski Berry and Murphy, 2004).

Sphingolipids species were also subjected to the analysis of characteristic fragments upon MS/MS experiments (Table 2). In negative ion mode, the quasi-molecular ion Cer(d18:1/24:1) appears at [M-H]-m/z 646.61. Upon CID, Cer(d18:1/24:1) yield an ion at m/z 628.4, corresponding to loss of water, followed by loss of formaldehyde (HCHO) to give m/z 616.6, which dissociates to m/z 406.4 and 390.4 by loss of the fatty acyl moiety as ketene (loss of C22H43CH = C = O) (Supplementary Figure 3). The m/z 628.4 ion also gives m/z 237.2 and 263.2 (long-chain base moiety, LCB) by elimination of the fatty acyl moiety as an amide (loss of C_17_H_33_CONH_2_, 281 Da). GlcCer(d18:1/24:0) and GlcCer(d18:1/24:1) were observed at m/z 810.68 and 806.66, respectively, were identified similarly (Supplementary Figure 4; Hsu and Turk, 2002).

In ESI-, (3′-sulfo)Galβ-Cer(d18:1/18:0) or C18 sulfatide and (3′-sulfo)Galβ-Cer[d18:1/18:0(2OH)] or C18-OH sulfatide were observed as quasi-molecular ion [M-H]^−^ whereas C24:1-OH sulfatide was detected as [M-H_2_O-H]^−^ ion. Upon CID (MS^E^ acquisition mode), m/z 97 and m/z 241 ions were observed corresponding to sulfate ion (${\text{HOSO}}_{3}^{-}$) and to 3-sulfogalactosyl moiety, respectively. The direct loss of the fatty acyl (FA 18:0) as a ketene from m/z 822.54 (C18-OH sulfatide) via the NH-CO bond cleavage results in m/z 540 (LCB moiety), which undergoes a water loss to yield m/z 522 ion. The FA moiety for C18 sulfatide, C18-OH sulfatide and C24:1-OH sulfatide were recorded at m/z 308.2, 324.2, and 388, respectively (Hsu and Turk, 2004).

**Figure 3:**
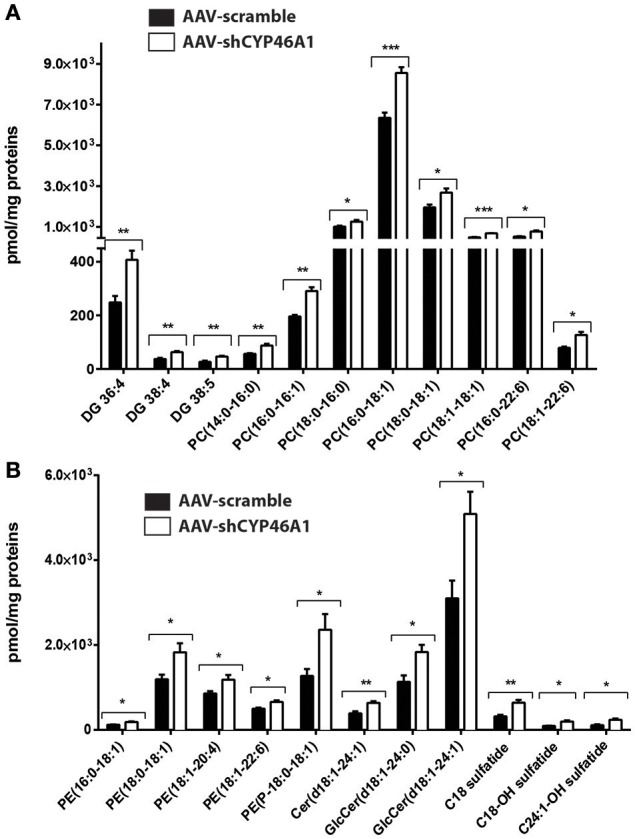
Relative quantification of lipids in hippocampus of C57Bl/6 mice after injection of AA V5-scramble (control) and AA V5-shCYP46A1. **(A,B)** Rise of phosphatidylcholine (PC), diacylglycerol (DAG), ceramides (Cer), sulfatide, phosphatidylethanolamine plasmalogen (PE-P) and phosphatidylethanolamine (PE) in AAV-shCYP46A1 hippocampus compared to the control. The results are expressed as nmol per mg of proteins and as the mean ± SD (*n* = 5 mice per group). Measurements were performed in triplicate. Unpaired *t*-test was performed. ^*^P < 0.05, ^**^P < 0.01, and ^***^P < 0.001.

Semi-quantitative ESI-HRMS analysis of the lipid molecular species in AAV-scramble and AAV-shCYP46A1 hippocampus tissues is presented in Figures 3A,B. Upon cholesterol accumulation, an increase of long-chain polyunsaturated fatty acids was observed. Phospholipids (PE, PE-P, PC) consisted mostly of long-chain fatty acid residues with 16 or 18 carbons and few very long-chain fatty acid residues (20 or 22 carbons) mainly with one unsaturation and in a less extend with 4 up to 6 double bonds (Figures 3A,B). AAV-shCYP46A1 injection induced an increase of DG species with medium chain length (36–38 carbons) and 4–5 double bonds (Figure 3A). An increase of GlcCer(d18:1/24:1), GlcCer(d18:1/24:0) and Cer(d18:1/24:1) were observed. For sulfatide species, an increase in C18 sulfatide, followed by C18-OH and C24:1-OH sulfatide were noticed (Figure 3B).

### Cholesterol accumulation resulting to *Cyp46a1* gene inhibition leads to increased PC biosynthesis

**Figure 4:**
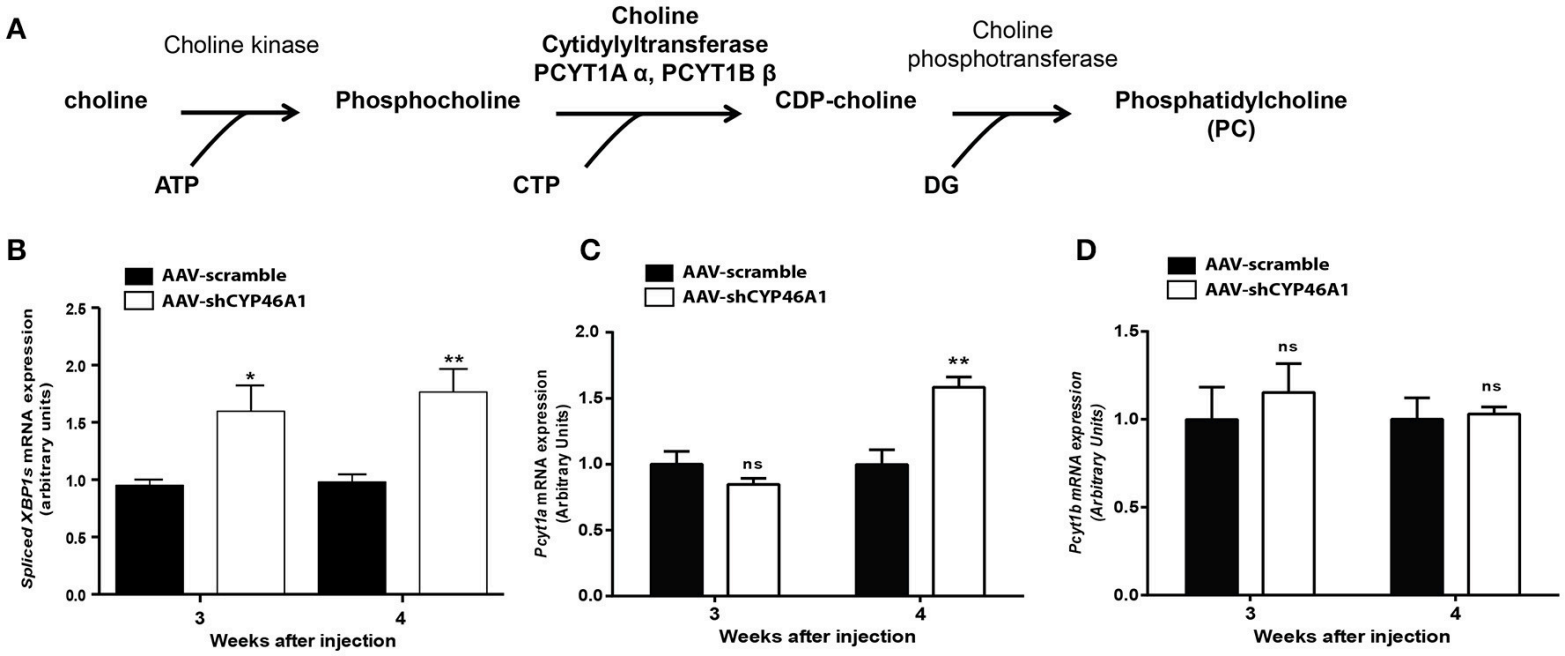
*De novo* synthesis of phosphatidylcholine *via* the Kennedy pathway **(A)**. Quantitative expression of spliced XBP1 form **(B)** and choline-phosphate cytidylyltransferase α (PCYT1A) **(C)** and choline-phosphate cytidylyltransferase β (PCYT1B) **(D)** genes after injection of AAV5-scramble and AAV-shCYP46A1 vectors in the hippocampus of C57BL/6 mice. Quantitative expression of murine spliced XBP1 **(B)**, Pcyt1a **(C)** and Pcyt1b **(D)** genes were performed after 3 and 4 weeks post-injection (*n* = 5 mice per vector, per time). Expression data are normalized to the expression of AAV-scramble. Unpaired *t*-test was performed. ^*^P < 0.05; ^**^P < 0.01; ns, non-significant.

We observed by an untargeted approach an increase of PC species in AAV-shCYP46A1 injected hippocampus. We were wondering if this increase of PC species was due to an activation of the biosynthetic pathway. PC is primarily synthetized by the citydine diphosphocholine (CDP) pathway (Kennedy pathway). After phosphorylation of choline into phosphocholine, choline cytidylyltransferase converts phosphocholine to CDP-choline in the presence of Cytidine Triphosphate (CTP) (Figure 4A). Phosphocholine moiety is then transferred to CDP-choline to diacylglycerol, producing PC (Fagone and Jackowski, 2013).

*In vitro* data show a relation between the expression of spliced XBP1 form and the choline phosphate cytidylyltransferase (Sriburi et al., 2004, 2007). Spliced XBP1 form is involved in ER stress previously described in our model (Djelti et al., 2015). Thus, we monitored the expression of gene encoding spliced XBP1 form and the two isoforms, α (*Pcyt1a*) and β (*Pcyt1b*) of the choline phosphate cytidylyltransferase by RT-qPCR (Figures 4B–D). We focus our attention on the mRNA expression of choline cytidyltransferase because it's the rate-limiting enzyme required for the synthesis of PC through the CDP-choline pathway (Pelech and Vance, 1984; Pelech et al., 1984). The expression of spliced *XBP1* gene, was increased by 1.5-fold, at 3 and 4 weeks after injection of AAV-shCYP46A1 vector (Figure 4B). In parallel, a 1.5-fold increase of *Pcyt1a gene expression* was evidenced 4 weeks after injection (Figure 4C). No increase of *Pcyt1b* was observed 3 and 4 weeks after injection (Figure 4D). The increase of *Pcyt1a* gene expression suggests that the accumulation of cholesterol resulting of CYP46A1 inhibition induced the expression of spliced XBP1 form and the increase of PC biosynthesis, 4 weeks after injection.

### Neuronal cholesterol accumulation induced imbalance in sphingolipid metabolism

**Figure 5:**
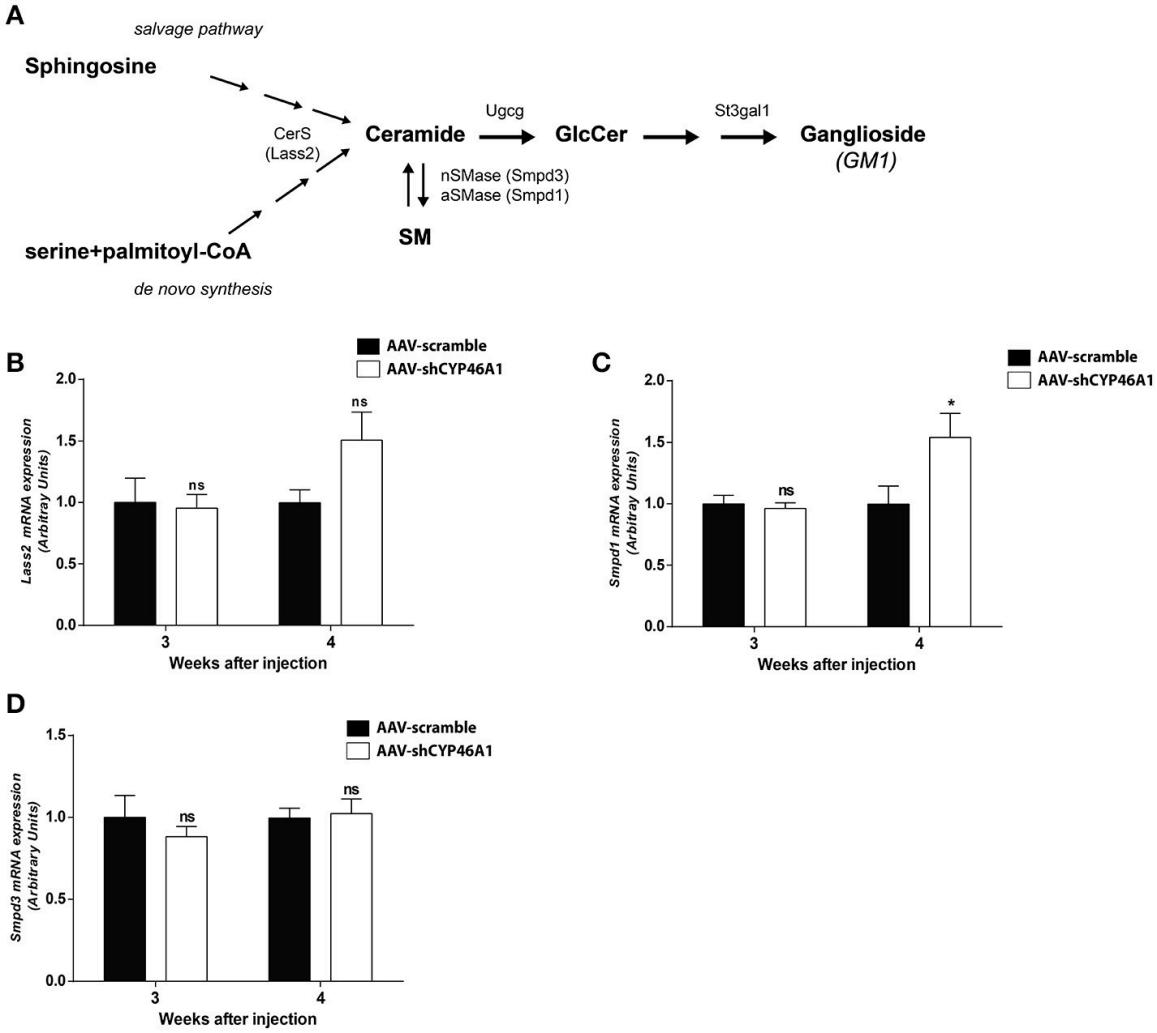
Sphingolipid metabolism **(A)**. Quantitative expression of ceramide synthase 2 (LASS2) **(B)**, acid sphingomyelinase (SMPD1) **(C)** and neutral sphingomyelinase (SMPD3) **(D)** genes in C57BL/6 mice after cerebral injections of AAV-scramble and AAV-shCYP46A1 vectors. Quantitative expression of murine *Lass2* (B), *Smpd1* (C) and *Smpd3* (D) genes were performed 3 and 4 weeks post-injection (*n* = 5 mice). Expression data are normalized to the expression of AAV5-scramble (control). Unpaired *t*-test was performed. ^*^P < 0.05; ns, non-significant.

Lipidomics analysis revealed an increase of ceramides and glucosylceramides species with long-chain fatty acids, namely Cer(d18:1/24:1), GlcCer(d18:1/24:0) and GlcCer(d18:1/24:1). No increase in sphingomyelin species was observed. To get further insight into the mechanism of ceramide increase, we investigated the expression of enzymes involved in ceramide metabolism. Ceramides are mostly generated by *de novo* synthesis from serine and palmitoyl-CoA. Ceramides are also produced by hydrolysis of sphingomyelin or by sphingosine from the salvage pathway (Figure 5A). The salvage pathway re-utilizes long chain sphingoid bases to form ceramide, through the action of ceramide synthase (Lass2) (Hannun and Obeid, 2008). LASS2 synthesizes ceramide species containing mainly long-chain fatty acyl moieties (22 up to 24 carbons), while synthesis of medium-chain ceramides (16 and 18 carbons) is relatively marginal (Laviad et al., 2008). We observed a tendency of the expression of Lass2 mRNA to be increased (1.6-fold) from 4 weeks after injection of AAV-shCYP46A1 (Figure 5B). Expression of *Smpd1* and *Smpd3* genes, coding, respectively for the acid sphingomyelinase and the neutral sphingomyelinase enzymes catalyzing the conversion of sphingomyelin into ceramide was then investigated. A 1.6-fold increase of *Smpd1* gene expression was observed 4 weeks after injection (Figure 5C) while no change was observed in *Smpd3* gene expression (Figure 5D). The increase of ceramides seems to result from the acid hydrolysis of sphingomyelin by the acid sphingomyelinase, SMPD1.

**Figure 6:**
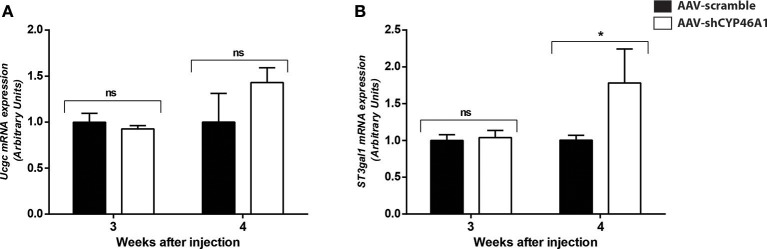
Quantitative expression of UDP-glucose ceramide glucosyltransferase (UCGC) **(A)** and ST3 beta-galactoside alpha-2,3-sialyltransferase (ST3GAL1) **(B)** after hippocampal injections of AAV-scramble and AAV-shCYP46A1 vectors. Quantitative expression of murine *Ucgc* **(A)**, *St3gal1* **(B)** genes were performed 3 and 4 weeks post-injection (*n* = 5 mice). Expression data are normalized to the expression of AAV-scramble (control). Unpaired *t*-test was performed. ^*^P < 0.05; ns, non-significant.

An increase in GlcCer species was also observed in AAV-CYP46A1 injected mice. We thus investigated the expression level of the enzymes involved in their synthesis. GlcCer is synthetized from ceramides (Figure 5A). A glucose residue is transferred to Cer by the UDP-glucose ceramide glucosyltransferase (UGCG) enzyme to form GlcCer. Addition of galactose to GlcCer results in the formation of lactosyl ceramide (LacCer), which is the precursor of the glycolipids also called gangliosides. Gangliosides are found on the surface of essentially all mammalian cells but are particularly abundant on neuronal cell surfaces like for instance GM1. Further addition of different sugars moieties (galactose, N-acetylgalactosamine, N-acetylglucosamine, sialic acid) in different configurations generates large numbers of gangliosides (Kolter et al., 2002; Yu et al., 2004). The biosynthetic pathway of the gangliosides is controlled by a few key glycosyltransferases and sialyltransferases. One of these reactions, is mediated by the transfer of sialic acid from CMP-sialic acid to galactose-containing substrates, to form GM1. As shown in Figure 6A, the expression of Ugcgc gene revealed no significant changes 3 or 4 weeks after injection of the AAV-shCYP46A1 vector. However, the expression of St3gal1 gene involved in conversion of lactosylceramide in gangliosides was increased 4 weeks after injection only (Figure 6B).

### Increased level of the lysosomal protein LAMP-1 and accumulation of phagolysosomes after inhibition of *Cyp46a1* gene expression

**Figure 7:**
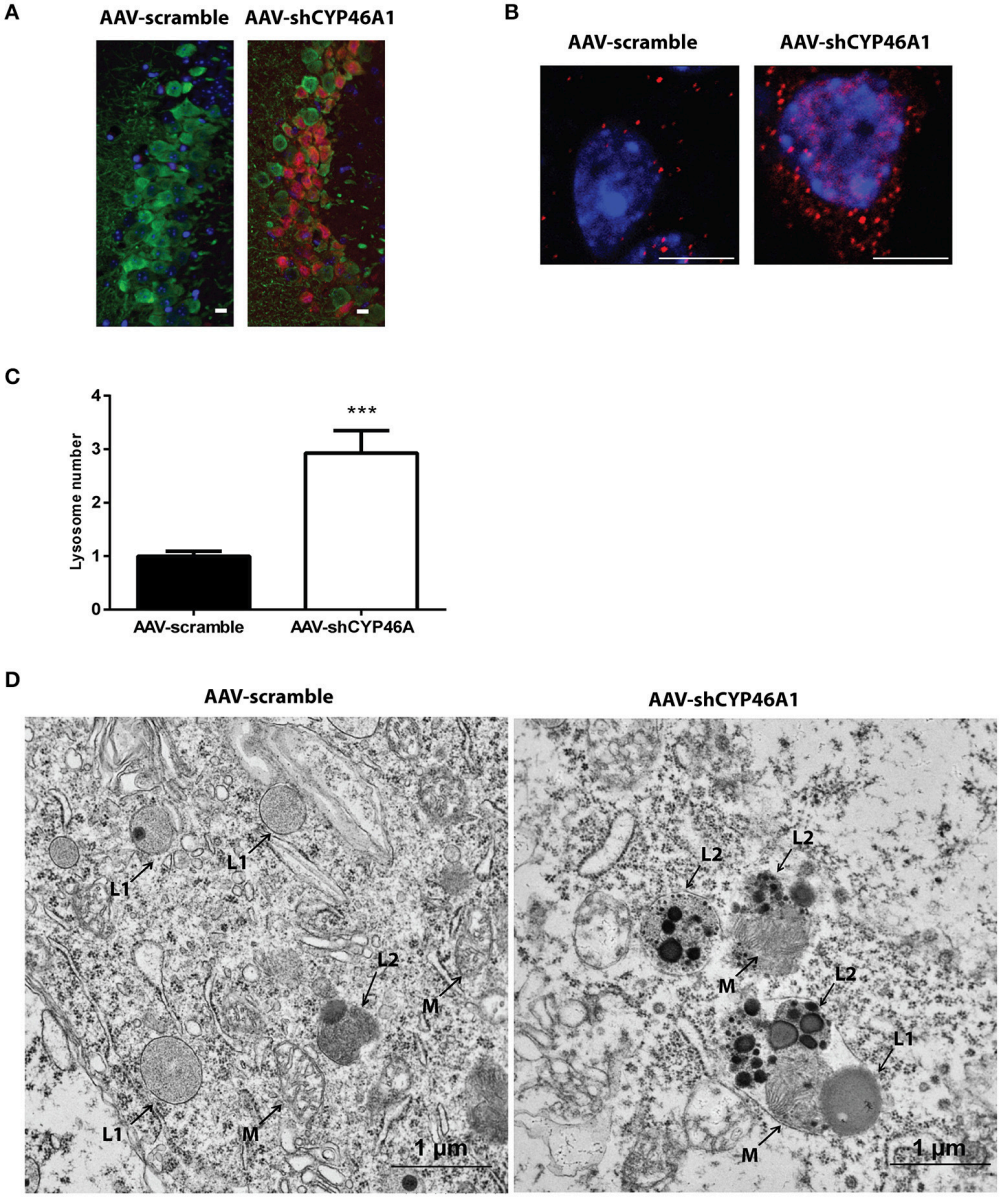
Accumulation and ultrastructural modifications of lysosomes in CA3a neurons of AAV-shCYP46A1 injected mice. **(A)** Representative eGFP immunostaining (green) and LAMP-1 immunoreactivity (lysosomal cell, red) (scale bar: 50 μm) **(B)** Representative image by laser confocal microscopy showing increased puncta-like immunoreactivity of LAMP-1 (red) in CA3a neurons of the hippocampus of C57BL/6 mice 4 weeks after injection of AAV-shCYP46A1 vector compared to AAV-scramble. Nuclei are counterstained with DAPI (blue) (scale bar: 6 μm). **(C)** Quantification of the number of LAMP-1-positive lysosomes in CA3a pyramidal cells at 4 weeks after AAV5-shCYP injection normalized to values from AAV5-scramble injected mice (30 cells of 5 mice per vector per time; 2-tailed unpaired *t*-test was performed. ^***^P < 0.0001. **(D)** Electron micrographs showing abnormal lysosomes in CA3a neurons of C57BL/6 mice 4 weeks after injection of AAV-shCYP46A1 vector (scale bar: 1 μm). (M): mitochondria, (L1): primary lysosomes, (L2): secondary lysosomes.

Late endosomal cholesterol accumulation leads to impaired intra-endosomal trafficking (Sobo et al., 2007). In our previous study, we found that endosomal trafficking was disturbed in CA3a pyramidal cells of mice injected with AAV-shCYP46A1 vector leading to enlarged endosomes (Djelti et al., 2015). To confirm that neuronal cholesterol accumulation in AAV-shCYP46A1 injected animals leads to impaired endosomal trafficking, we investigated lysosome number and morphology. First, we observed that the immunoreactivity of the lysosomal-associated membrane protein 1 (LAMP-1) was increased together with a 2.9 ± 0.42-fold increase in lysosome numbers in neurons of AAV-shCYP46A1 injected mice compared to the control (AAV-scramble) (Figures 7A,B). Ultrastructural analysis revealed abnormal lysosomes in CA3a neurons of mice injected with AAV-shCYP46A1, 4 weeks after injection (Figure 7C). In neurons of AAV-scramble injected mice, the arrows represent mitochondria (M), primary lysosomes (L1) and secondary lysosomes (L2) with normal morphology and homogenous with finely granular content (Figure 7C-left panel). In contrast, in neurons of AAV-shCYP46A1 injected mice, electron micrograph exhibits autophagolysosomes containing dark content with numerous electron-dense vesicles and mitochondria in the lumen (Figure 7C-right panel). Altogether, these observations suggest that cholesterol accumulation in neurons leads to an impairment of the endosomal-lysosomal membrane trafficking.

## Discussion

**Figure 8:**
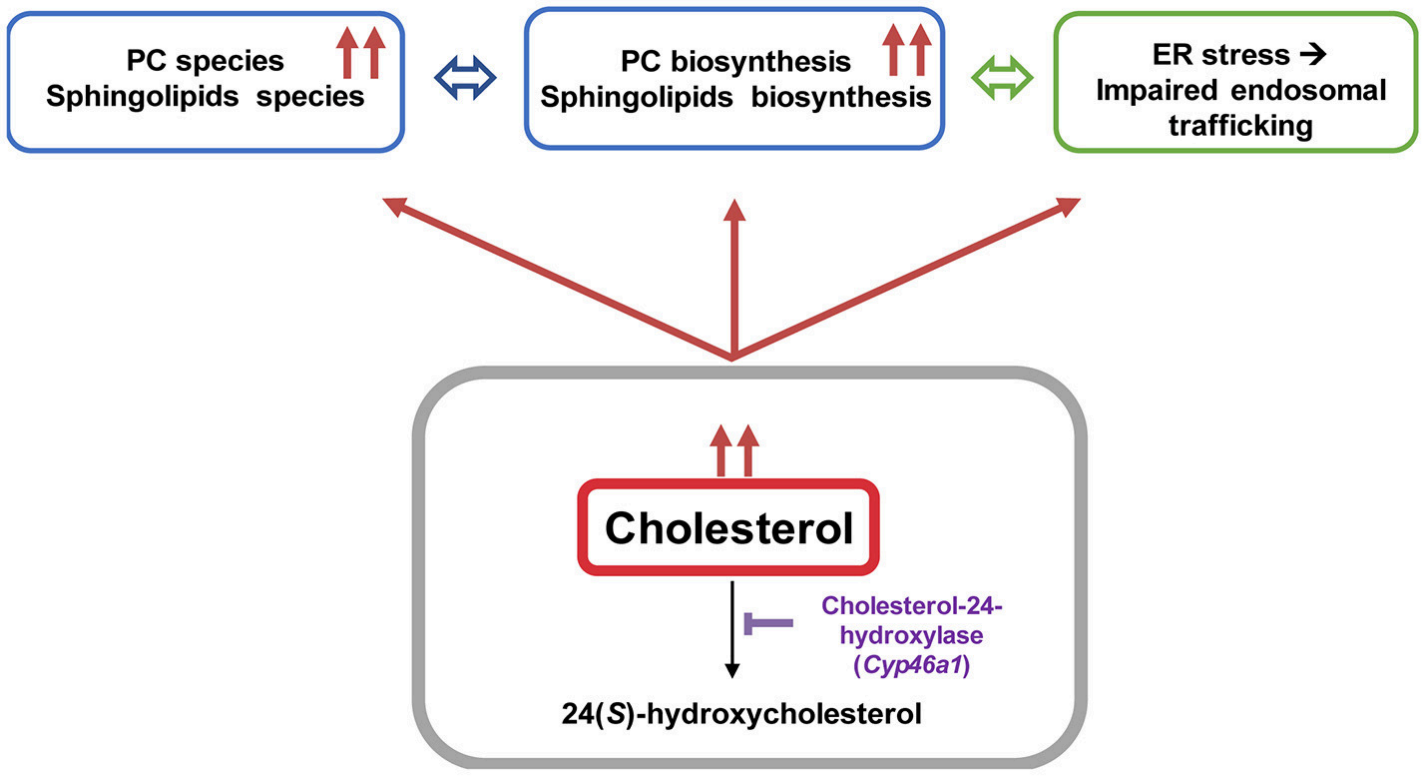
Cholesterol overload in neurons induced by Cyp46a1 down-regulation in mice hippocampus disrupts lipid metabolism and lysosomal trafficking.

Impairment in cholesterol metabolism is involved in many neurodegenerative diseases including AD (Anstey et al., 2008). However, the molecular mechanism that stands behind altered cholesterol levels and neurodegeneration remains incompletely understood. We established an *in vivo* mouse model to bring out the consequences of lipid perturbation associated with a specific cholesterol accumulation in neurons by down-regulating *in vivo* the cholesterol-24 hydroxylase coding gene (*cyp46a1*) expression using an AAV5-based RNA interference strategy (Djelti et al., 2015; Figure 8). The neuronal cholesterol-24 hydroxylase enzyme is involved in the cerebral clearance of cholesterol by catalyzing the conversion of cholesterol into 24(*S*)-hydroxycholesterol and, to a lesser extent, 25-hydroxycholesterol (Lund et al., 1999, 2003). The synthesis of 24(*S*)-hydroxycholesterol and its secretion from the brain represent the main mechanism of cholesterol turnover in this organ (Lund et al., 2003).

Our previous study showed that inhibition of *cyp46a1* gene expression in wild-type mice lead to abnormal processing of APP with Aβ peptides accumulation and tau hyperphosphorylation, two hallmarks of AD, associated with ER stress and enlargement of the endosomal/lysosomal compartments. This cascade of events finally caused apoptotic neuronal death leading to cognitive impairment and hippocampal atrophy. These toxic consequences of cholesterol increase were strongly aggravated in APP23 mice (Djelti et al., 2015).

The aim of the present study was to characterize the detailed lipid perturbations associated with cholesterol accumulation induced by CYP46A1 inhibition in hippocampal neurons. Lipid modifications were analyzed 4 weeks after injection of an AAV5 vector expressing a short-hairpin (sh) RNA against *cyp46a1* gene, a time where neuronal loss was shown to appear in this model (Djelti et al., 2015).

The delivery of *Cyp46a1* sh-RNA in the *stratum lacunosum moleculare* region of C57Bl/6 mice hippocampus using AAV5 vector stereotactic injection leads to an efficient neuronal inhibition of *Cyp46a1* gene expression in CA3a region in AAV-shCYP46A1 injected mice (Chali et al., 2015). This inhibition leads to a decrease of 24(*S*)-hydroxycholesterol and 25-hydroxycholesterol content, accompanied by an accumulation of cholesterol.

Previous studies have shown that the cholesterol 24-hydroxylase knock-out mice (*Cyp46a1*^(−/−)^ exhibit 50% decrease in *de novo* cholesterol synthesis and a corresponding 50% decrease in cholesterol excretion from the brain without modification of the cholesterol content (Lund et al., 2003; Xie et al., 2003; Meljon et al., 2014). This shows that alterations of *Cyp46a1* gene expression at an early developmental stage are compensated by homeostatic adaptation of cholesterol metabolism, and in particular cholesterol synthesis. In our *in vivo* model, the cholesterol synthesis was not modified. Indeed, we did not monitor any changes in cholesterol precursors content, such as cholestanol, desmosterol or 5α,6α-epoxycholesterol. Moreover, expression of genes coding the enzymes involved in the synthesis of cholesterol or its regulation (HMG-CoA reductase and Srebp-1) remained unchanged in the hippocampus of injected mice (Djelti et al., 2015). The 70% inhibition of *Cyp46a1* gene expression by AAV-shCYP46A1 blocks the clearance of cholesterol leading to an accumulation of cholesterol with no effect on cholesterol biosynthesis (Djelti et al., 2015).

The lipid analysis carried out in the present study revealed an increase of brain structural glycerophospholipids (PC, PE, PE-P) and sphingolipids. PE, PE-P and PC species with medium-chain length fatty acyls were augmented in hippocampi with an accumulation of cholesterol as well as an increase of *Pcyt1*α expression, an enzyme involved in the rate-limiting step of PC biosynthesis. An *in vitro* study showed that free cholesterol accumulation in macrophages up-regulated PC synthesis (Sriburi et al., 2004, 2007). The increase of PC bulk is an adaptive response to prevent toxic effect of cholesterol excess by maintaining the ratio free cholesterol: PC in the membrane to a physiological level. Increase of PC was shown to be induced by ER stress. In our previous study, we showed in our model a rapid and major increase of ER stress (already present 3 weeks after injection) with phosphorylation of PERK and an increase of CHOP protein in response of neuronal accumulation of cholesterol.

Sphingolipids e.g., sphingomyelin, ceramides, sulfatides, gangliosides are enriched in the central nervous system. In addition to important structural role like membrane integrity, sphingolipids (ceramides or sphingosine-1-phosphate) in association with cholesterol function as second messengers to modulate a variety of signaling event. Perturbations in sphingolipid homeostasis and trafficking have been extensively documented in neurodegeneration disorders like Niemann-Pick type C or AD (He et al., 2010; Fan et al., 2013). The lipid profiling carried out in the present study also revealed that elevated species across multiple sphingolipid classes were increasing with cholesterol accumulation. Ceramides, glucosylceramides and sulfatides with medium or long-chain fatty acyls (C18; C24) were increased. Though, sphingomyelin content was not modified. Early reports show that ceramide levels are elevated at the earliest clinically recognizable stage of AD mediating oxidative stress-induced neuronal death (Cutler et al., 2004; He et al., 2010). Alterations of long-chain ceramides together with an increase of cholesterol were observed in brains of AD patients (Soreghan et al., 2003; Cutler et al., 2004; Han, 2005; Mattson et al., 2005).

Ceramide is generated either by the degradation of sphingomyelin *via* the action of sphingomyelinases or *via de novo* synthesis through the enzyme ceramide synthase, Lass2. Sphingomyelin degradation is achieved either by the acid sphingomyelinase, Smpd1 (lysosomal) or the neutral sphingomyelinase Smpd3 (membrane) to form ceramide. We observed that the expression of *Smpd1* gene encoding acid sphingomyelinase was increased while the expression of *Smpd3* gene encoding neutral sphingomyelinase and *Lass2* gene encoding ceramide synthase were not modified. This observation is in a perfect agreement with a previous study showing that the expression of several genes involved in sphingomyelin metabolism, are increased in AD brain including acid sphingomyelinase (Katsel et al., 2007).

The excess of neuronal cholesterol after injection of AAV5-shCYP46A1 conduced to an increase in gangliosides GM1. A significant increase expression of gene encoding ST3 β-galactoside α-2,3-sialyltransferase 1, ST3GAL1 was observed. Interestingly, several strong indications point toward an important role of gangliosides in AD pathogenesis. Indeed, β-amyloid peptides interact with GM1, which is abundantly expressed in neural cell membranes and pile in lipid rafts to form amyloid fibrils (Yamamoto et al., 2008; Ariga et al., 2011; Matsuzaki, 2011; Yanagisawa, 2011). Some studies reported that the concentration and composition of gangliosides are altered in the brains of AD patients and in transgenic mouse models of AD (Chan et al., 2012). In our previous study, we showed that neuronal cholesterol accumulation was associated with the enlargement of the endosomal/lysosomal compartments disturbing the processing of APP and leading to the accumulation of Aβ peptides (Djelti et al., 2015). Various studies have documented abnormal endosomal morphology in the brain of individuals with AD. Enlarged early endosomes were formed in most pyramidal neurons in the brains of patients with sporadic AD (Cataldo et al., 2000). *In vitro*, after cholesterol loading treatment, primary culture neurons exhibited enlarged endosomes (Cossec et al., 2010). Increase in number of secondary lysosomes and changes in levels of lysosomal enzymes have previously been also associated with brain aging and age-related neurodegeneration (Lynch and Bi, 2003). It has been also shown that the lysosomal membrane protein LAMP-1 was upregulated at both the mRNA and protein level in the AD brain (Barrachina et al., 2006). Our experiment revealed that the lysosomes were abnormal, swelling and their numbers increased. These observations confirmed previous results showing modification of lysosome morphology induced by cholesterol excess (Barrachina et al., 2006). To summarize, we showed that *in vivo* cholesterol accumulation in hippocampal neurons following inhibition of CYP46A1 enzyme leads to major dysregulation of lipid homeostasis with combined accumulation of different lipid species from PC, PE, ceramides, sulfatides and gangliosides classes (Figure 8). Such modifications of the lipidome were also identified in cerebral tissues of AD patients (Han et al., 2001, 2002; Cutler et al., 2004; Han, 2005; Chan et al., 2012). Moreover, *in vivo* cholesterol accumulation in neurons induced a cascade of events with amyloid pathology, tau hyperphosphorylation and ER stress leading to neuronal dysfunction and cell death. In conclusion, lipidomics analysis combined with RNA interference-based strategy thus provides a powerful tool to further elucidate the specific roles of lipid intermediates in cell signaling and pathology of neurodegenerative diseases.

## Author contributions

SAy, FD, SAl, MG, NA, PA, and NC conceived the experiments. FD performed AAV injections. SAy, AR, DD, MG, and NA performed MS experiments. FD, JV, and IB performed RT-PCR experiments. FD, SAl, and DL performed microscopy experiments. SAy, FD, SAl, NA, AR, and NC analyzed the data. SAy, FD, SAl, EH, NA, NC, and OL interpreted the experiments and wrote the paper.

### Conflict of interest statement

The authors declare that the research was conducted in the absence of any commercial or financial relationships that could be construed as a potential conflict of interest.

## Abbreviations

AD: Alzheimer's disease
AAV5: Adenovirus associated vector serotype 5
24-OHC: 24(S)-hydroxycholesterol
Cyp46a1: cholesterol-24(S)-hydroxylase gene
Aβ: amyloid peptide
APP: Amyloid precursor protein
BACE1: β-secretase
sh-CYP46A1: short hairpin RNAi CYP46A1
UPR: Unfolded Protein Response
UPLC: Ultra-performance liquid chromatography
PE: Phosphatidylethanolamine
PC: Phosphatidylcholine
Cer: Ceramide
GlcCer: Glucosylceramide
DG: Diacylglycerol
TBP: TATA-box binding protein gene
PCA: Principal Components Analysis
PLS-DA: Partial least square-Discriminant Analysis
OPLS-DA: Orthogonal Partial least square-Discriminant Analysis
QC: quality control
PCYT1α: choline cytilyltransferase α
PCYT1β: choline cytilyltransferase β
SMPD1: Sphingomyelin phosphodiesterase 1
SMPD3: Sphingomyelin phosphodiesterase 3
LASS2: ceramide synthase 2
ST3GAL1: ST3 β -galactoside α -2,3-sialyltransferase 1
UGCG: UDP-glucose ceramide glucosyltransferase
DAPI: 4′,6-diamidino-2-phenylindole
eGFP: enhanced green fluorescent protein
LAMP-1: lysosomal-associated membrane protein 1.
