# Supplementary material for: Neuronal Cholesterol Accumulation Induced by Cyp46a1 Down-Regulation in Mouse Hippocampus Disrupts Brain Lipid Homeostasis
Source: Front Mol Neurosci. 2017 Jul 11;10:211. doi: 10.3389/fnmol.2017.00211 (PMC5504187; doi:10.3389/fnmol.2017.00211)
Supplement: Supplementary file 1 [file Table1.PDF]

Supplementary Table S1: sh-RNA sequence used in this study

Name	Sense strand	Loop	Anti-sense strand	Length (nt)	Position
Scramble	**GTCTTCTAGATTTGTGAGAGGGAACTT**	TTGATATCCG	**AAGTTCCCTCTCACAAATCTAGAAGAC**	27	None
shCYP46A1	**GTATGGTCCTGTTGTAAGAGTCAATGT**	TTGATATCCG	**ACATTGACTCTTACAACAGGACCATAC**	27	210-236

nt: nucleotides
