# Supplementary material for: Neuronal Cholesterol Accumulation Induced by Cyp46a1 Down-Regulation in Mouse Hippocampus Disrupts Brain Lipid Homeostasis
Source: Front Mol Neurosci. 2017 Jul 11;10:211. doi: 10.3389/fnmol.2017.00211 (PMC5504187; doi:10.3389/fnmol.2017.00211)
Supplement: Supplementary file 2 [file Table2.PDF]

Supplementary Table S2: RT-qPCR sequences of oligonucleotide primers used in this study

Genes	Oligonucleotide	*Sequence*
Lass2	Upper primer	5' TCC CTC AGC TCA GAG TCC TCA CA 3'
	Lower primer	5' CAC ATG CAG CAC CTC GCC TAC 3'
Pcyt1a	Upper primer	5' AGC GTT GCG CAG TTG GTT TAC 3'
	Lower primer	5' CCT GCA GGC TTC TTC CAT AGT CA 3'
Pcyt1b	Upper primer	5' CAC AGC CTC GAC TGA CCC TGA 3'
	Lower primer	5' TTT TTC ATG AGG TGC TTG GCA CT 3'
Smpd1	Upper primer	5' GCC TTT GGG TGG CAG AAC CT 3'
	Lower primer	5' CCA CAT TGG GCT CCT TCT TCA G 3'
Smpd3	Upper primer	5' TGT CAT GGA CGT GGC CTA TCA 3'
	Lower primer	5' CTT CCC ACC TGC ACC TTG AGA 3'
ST3gal1	Upper primer	5' GGT ACA CCC GAA CCC AGC AC 3'
	Lower primer	5' CAA CCA ACG TGC ACT TGC AGA 3'
Ugcg	Upper primer	5' TCA TGG TCT TCT TCA TGT GCC ACT 3'
	Lower primer	5' ACA CAG TGT GCC ACC CTG GAC 3'
