# Supplementary material for: Neuronal Cholesterol Accumulation Induced by Cyp46a1 Down-Regulation in Mouse Hippocampus Disrupts Brain Lipid Homeostasis
Source: Front Mol Neurosci. 2017 Jul 11;10:211. doi: 10.3389/fnmol.2017.00211 (PMC5504187; doi:10.3389/fnmol.2017.00211)
Supplement: Supplementary file 3 [file Presentation1.PDF]

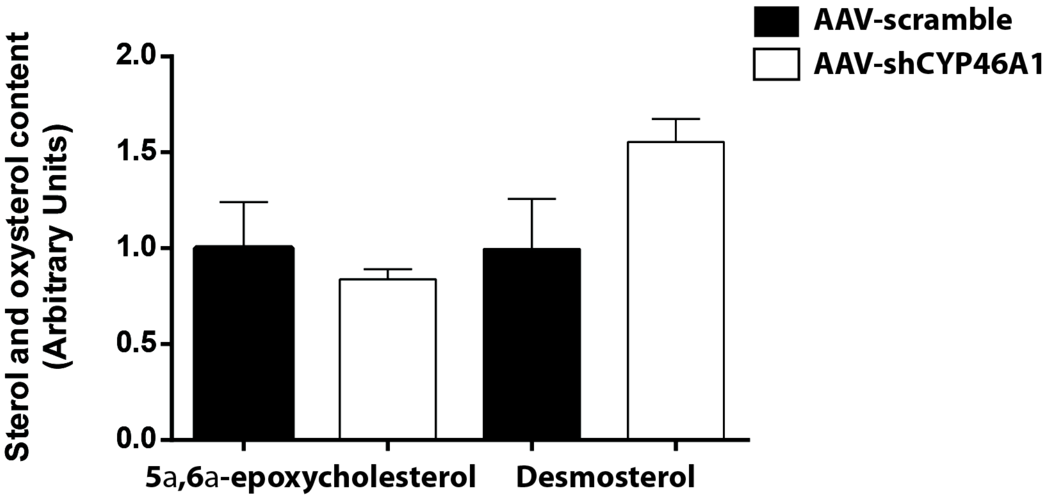

Supplementary Figure 1. Measurement of 5α,6α-epoxycholesterol and desmosterol after AAV-shCYP46A1 hippocampal injection. AAV-scramble (control) or AAV-shCYP46A1 vector was injected in the *stratum lacunosum moleculare* of hippocampus in C57BL/6J mice. Sterols were extracted, derivatized and analyzed by UPLC-ESI-Q-TOF in MS scan mode. 5α,6α-epoxycholesterol and desmosterol contents were quantified and normalized to AAV-scramble content (n= 5 mice).
