# Supplementary material for: Neuronal Cholesterol Accumulation Induced by Cyp46a1 Down-Regulation in Mouse Hippocampus Disrupts Brain Lipid Homeostasis
Source: Front Mol Neurosci. 2017 Jul 11;10:211. doi: 10.3389/fnmol.2017.00211 (PMC5504187; doi:10.3389/fnmol.2017.00211)
Supplement: Supplementary file 4 [file Presentation2.PDF]

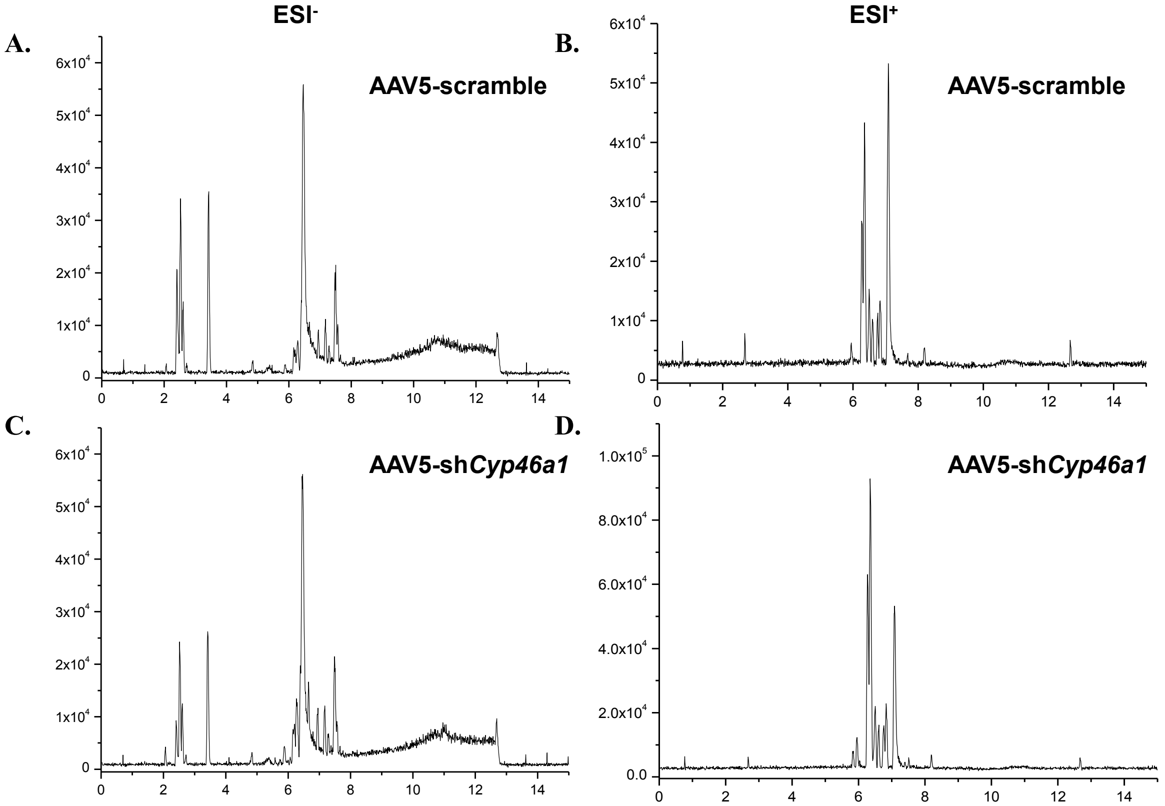

Supplementary Figure 2. Chromatograms of control (AAV-scramble) and AAV-shCYP46A1 lipid extracts acquired in negative (A-C) and positive ion mode (B-D).
