# Supplementary figures and images for: Neuronal Cholesterol Accumulation Induced by Cyp46a1 Down-Regulation in Mouse Hippocampus Disrupts Brain Lipid Homeostasis
Source: Front Mol Neurosci. 2017 Jul 11;10:211. doi: 10.3389/fnmol.2017.00211 (PMC5504187; doi:10.3389/fnmol.2017.00211)

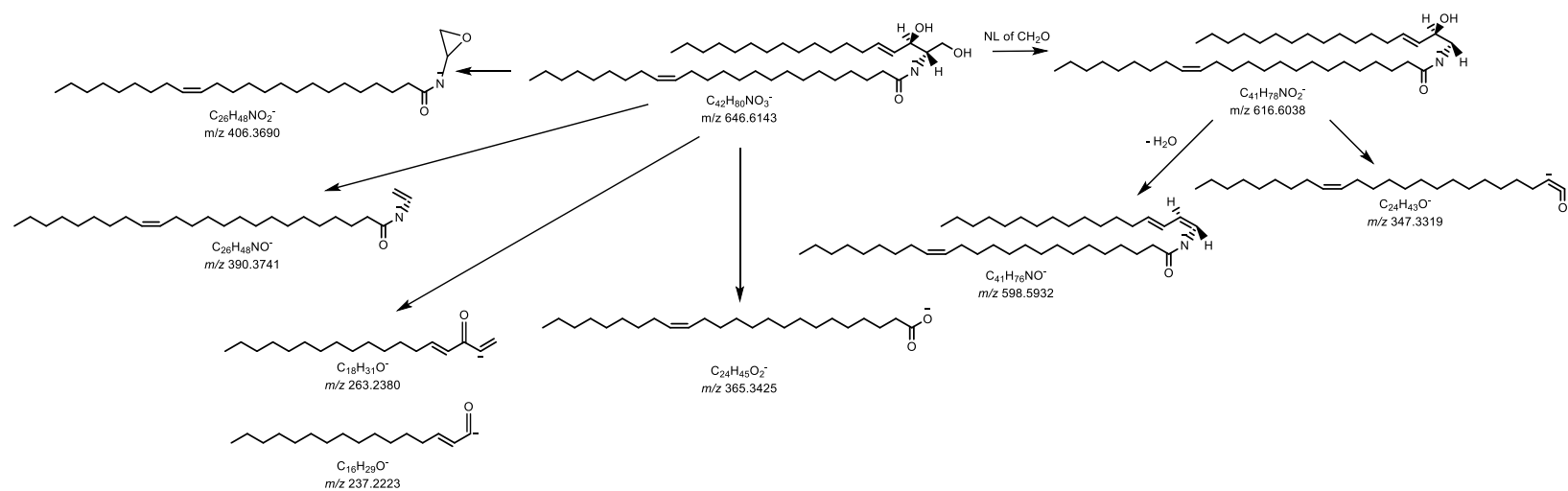

**Supplementary Figure 3.** Fragmentation of Cer(d18:1/24:1). tR=7.76, [M-H]<sup>-</sup>  $m/z$  646.61

Supplement: Supplementary file 5 [file Presentation3.PDF]

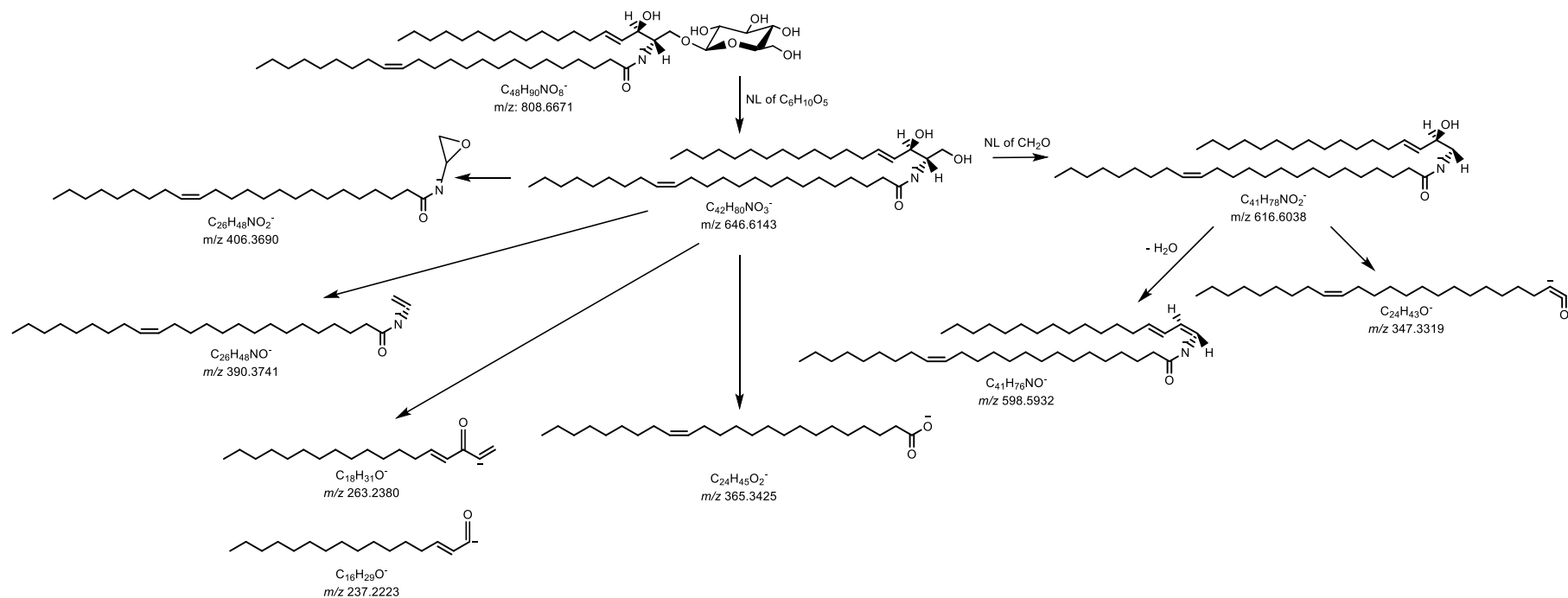

**Supplementary Figure 4.** Fragmentation of GlcCer(d18:1/24:1). tR=7.28, [M-H]<sup>-</sup> m/z 808.66

Supplement: Supplementary file 6 [file Presentation4.PDF]
